# Thrombin Generation in Pediatric Haemophilia A Patients on Extended Half‐Life FVIII versus Non‐FVIII Therapies

**DOI:** 10.1111/hae.70258

**Published:** 2026-03-12

**Authors:** Jessica Garcia, Sean G. Yates, Ravi Sarode, Guy Young, Ayesha Zia

**Affiliations:** ^1^ Department of Pediatrics Division of Hematology/Oncology UT Southwestern Medical Center Dallas Texas USA; ^2^ Children's Health Dallas Texas USA; ^3^ Department of Pathology UT Southwestern Medical Center Dallas Texas USA; ^4^ Hemostasis and Thrombosis Center, Cancer and Blood Diseases Institute, Children's Hospital Los Angeles, University of Southern California Keck School of Medicine Los Angeles California USA

**Keywords:** efanesoctocog alfa, emicizumab, FVIII, haemophilia, thrombin generation

## Abstract

**Introduction:**

The therapeutic landscape for patients with haemophilia A (PwHA) is rapidly evolving with the introduction of extended half‐life FVIII (EHL‐FVIII) and non‐FVIII therapies that mimic FVIII, such as emicizumab (EMI). Monitoring non‐factor therapies in the laboratory poses challenges; however, the thrombin generation assay (TGA) can be utilized to evaluate hemostatic capacity.

**Aim:**

To compare the endogenous thrombin potential (ETP) and peak thrombin (PT) in pediatric patients with moderate to severe haemophilia A (SHA) undergoing EHL‐FVIII therapies and EMI.

**Methods:**

Platelet‐poor plasma (PPP) from PwHA on EHL‐FVIII or EMI prophylaxis was tested on the calibrated automated thrombogram (CAT) using PPP low reagent.

**Results:**

ETP and PT were significantly higher in the EHL‐FVIII group compared to the EMI group.

**Conclusion:**

Pediatric patients on EHL‐FVIII prophylaxis demonstrated higher ETP in vitro using PPP compared to those on EMI prophylaxis. These findings highlight the need for further systematic investigations to explore the implications of these differences in bleed control.

## 1 Introduction

Haemophilia A is an X‐linked bleeding disorder due to a deficiency in factor VIII (FVIII). Prophylaxis to reduce and prevent long‐term bleeding complications, such as hemophilic arthropathy, is recommended in patients with severe or moderate haemophilia and severe phenotypes. [1, 2]. Historically, the management of persons with haemophilia A (PwHA) was focused on intravenous factor replacement, initially using cryoprecipitate and, later, plasma‐derived and recombinant FVIII concentrates, requiring frequent intravenous infusions [3]. The therapeutic landscape for PwHA is rapidly evolving with extended half‐life FVIII (EHL‐FVIII) and non‐FVIII therapies, which mimic FVIII, such as EMI. Current guidelines recommend maintaining a FVIII trough level of 3%–5% to prevent bleeds [2]. EHL‐FVIII and non‐FVIII therapies are considered standard of care for PwHA, and each maintains FVIII or FVIII equivalence levels at the recommended level; however, head‐to‐head comparative data for EHL‐FVIII and non‐FVIII therapies are lacking.

Laboratory monitoring of novel non‐factor therapies is challenging as they do not replace deficient factor. Thrombin generation assay (TGA) is a testing modality that has the potential to assess the hemostatic capacity of novel therapies and tailor treatment to specific needs. Although the TGA is not yet utilized in routine clinical care, it can measure the rate and amount of thrombin generation, thereby evaluating the overall hemostatic capacity. Current evidence reports ability to distinguish between milder phenotypes of PwHA despite similarly low levels of FVIII [4]. Additionally, studies have shown that factor dosing guided by endogenous thrombin potential (ETP), a key parameter obtained from TGA, led to a reduction in annualized bleeding rates (ABR) [5]. The ability to assess hemostatic potential to guide treatment has implications for improving patient management and the development and validation of emerging haemophilia therapies. Thrombin generation data comparing novel FVIII and non‐FVIII therapies remain critically insufficient in pediatric patients with haemophilia A (PwHA). Addressing this deficiency is essential in optimizing individualized treatment and ensuring informed clinical decision‐making. We hypothesized that thrombin generation would be higher in patients receiving EHL‐FVIII products compared to EMI.

## 2 Materials and Methods

### 2.1 Study Population

We enrolled pediatric PwHA on prophylaxis with EMI or EHL‐FVIII therapies receiving care at the haemophilia treatment center at UT Southwestern, Dallas, Texas (IRB STU 122016–026) between 2021 and 2025. We included consecutive PwHA with moderate and severe haemophilia on novel therapies during this period. Patients on EMI were either on 1.5 mg/kg weekly, or 3 mg/kg every 2 weeks. Patients on adynovate were on twice weekly, and those on Efanesoctocog alfa were on once weekly. We excluded patients with mild haemophilia, those with haemophilia A and concomitant bleeding disorders, haemophilia patients with thrombophilia or a history of thrombosis, on anticoagulation or anti‐platelet agents.

### 2.2 Clinical Data Collection

Patient information was collected at the time of blood collection. The variables recorded included age, sex, race, body mass index (BMI), and haemophilia severity. Normal weight was defined as a BMI between the fifth and 85^th^ percentiles; overweight as a BMI >85^th^ percentile to <95^th^ percentile; and obese as a BMI ≥95^th^ percentile adjusted for sex and age.

### 2.3 Laboratory Collection

Blood was collected in 3.2% sodium citrate tubes and spun at 3000 g for 15 min within 30 min of collection. The supernatant was collected and transferred to a second spin tube and centrifuged at 3000 g for 15 min, yielding platelet‐poor plasma (PPP). Plasma samples were then aliquoted and stored at −80°C until testing.

### 2.4 Laboratory Assays

#### 2.4.1 TGA

Samples were thawed in a 37°C water bath for 5 min immediately before performing thrombin generation testing. The reaction was triggered with the TG assay PPP low reagent (STAGO). All patient samples were run in triplicate. Thrombin generation was measured using the calibrated automated thrombogram (CAT, STAGO) in a Fluoroskan Ascent VR fluorometer equipped with a dispenser, as published previously [6]. Briefly, 80 microliters of PPP were dispensed into the wells of round‐bottom 96‐well microtiter plates. Twenty microliters of PPP reagent were added to the plasma samples. The starting reagent FLUCA Kit containing fluorogenic substrate and CaCl2 were automatically dispensed (20 microliters/well) by the fluorometer. A dedicated software program, ThrombinoscopeVR (version 3.0.0.29) was used to calculate thrombin activity against the calibrator and display thrombin activity versus time. The parameters obtained included lag time (min), peak thrombin (PT) (nM), time to peak (min) and ETP (area under the curve, nM.min).

### 2.5 FVIII Activity

All samples from patients receiving EHL‐FVIII were obtained at trough, except from one patient for whom a random sample was collected 1 day before infusion. FVIII activity was measured using a one‐stage clot‐based assay on the Siemens CS‐5100 analyzer (Siemens Healthineers, Erlangen, Germany). PPP samples were mixed with FVIII‐deficient plasma, and clotting was initiated using Dade Actin FSL reagent for adynovate samples and STA‐PTT reagent for efanesoctocog alfa samples. Calcium chloride was then added to initiate clot formation. Clotting time was recorded, and FVIII activity (%) was calculated based on a calibration curve generated from serial dilutions of reference plasma with assigned FVIII concentrations. All testing was performed according to the manufacturer's instructions and standard laboratory protocols.

### 2.6 Statistical Analysis

We summarized demographic and clinical characteristics as means or proportions when appropriate. Thrombin generation data was compared between patients receiving EMI and those on EHL‐FVIII using an unpaired *t*‐test. Normality of the data was confirmed via the Shapiro‐Wilk test (p.0.05), allowing for parametric analysis. Statistical significance was determined at *p* < 0.05, and all calculated *p* values were 2‐ sided. Data are presented as mean ± standard deviation (SD). GraphPad Prism version 10 was used.

## 3 Results

### 3.1 Entire Cohort

A total of 23 consecutive male participants met the inclusion criteria for the final analysis, ranging in age from 1.8 to 18.1 years (mean age: 10.74 years; SD: 4.90). Of these, 20 (87%) had severe haemophilia A (SHA), while 3 (13%) had moderate haemophilia A (MHA). At the time of TGA collection, 7 participants (30%) were classified as overweight or obese.

### 3.2 EHL‐FVIII Cohort

This subgroup included 11 male participants, of whom 3 (27%) had MHA and 8 (73%) had SHA. Two participants contributed samples at two distinct time points, each while receiving a different EHL‐FVIII therapy. Ages at sample collection ranged from 4.05 to 18.07 years (mean: 12.96; SD: 3.55). Four participants (36%) were classified as obese. At the time of collection, all patients were receiving either Adynovate or efanesoctocog alfa (Table 1).

**TABLE 1:** Characteristics of PwHA with and without inhibitor on emicizumab or EHL‐FVIII prophylaxis.

Variable	EMI prophylaxis (*N* = 12)	EHL‐FVIII prophylaxis (*N* = 13)
Mean age years (range)	8.34 (1.87–16.35)	12.96 (4.05–18.07)
**Race**		
Asian	1	0
Black	0	3
Caucasian	4	4
Hispanic	7	6
**Gender**		
Female	0	0
Male	12	13
**Type HA**		
Severe	12	9
Moderate	0	4
Mild	0	0
History of inhibitor	3	0
**BMI**		
Underweight	2	2
Normal	6	4
Overweight	0	1
Obese	3	6
N/A	1	0
Adynovate prophylaxis	0	4
Efanesoctocog alfa prophylaxis	0	9

Abbreviation: EHL, extended half‐life; N, number; PwHA, persons with haemophilia A.

### 3.3 EMI Cohort

Twelve male participants with SHA were included, ranging in age from 1.87 to 16.35 years (mean: 8.34; SD: 5.17). Three participants (25%) had a history of FVIII inhibitor and were classified as obese (Table 1).

### 3.4 TGA and FVIII

We analyzed thrombin generation in 25 samples from 23 pediatric patients with moderate (*n* = 3) to severe (*n* = 20) haemophilia A who were receiving either prophylactic EMI or EHL‐FVIII therapy. The EMI group included 12 patients (mean age 8.34 years, SD 5.17), while the EHL‐FVIII group included 13 samples from 11 patients (9 on efanesoctocog alfa and 4 on Adynovate; mean age 12.96 years, SD 3.55). FVIII activity levels were measured in all 13 EHL‐FVIII samples.

**FIGURE 1:**
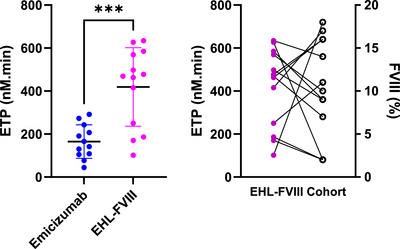
Endogenous thrombin potential is higher with EHL‐FVIII compared to emicizumab. A). Dot plot comparing endogenous thrombin potential (ETP, nM‐min) between extended half‐life factor VIII (EHL‐FVIII) and emicizumab in PwHA. ETP values were significantly higher in the EHL‐FVIII group (***p < 0.001), signifying greater thrombin generation capacity. B). Scatter plot showing the relationship between ETP (nM.min) and FVIII activity (%) within patients on EHL‐FVIII prophylaxis.

**FIGURE 2:**
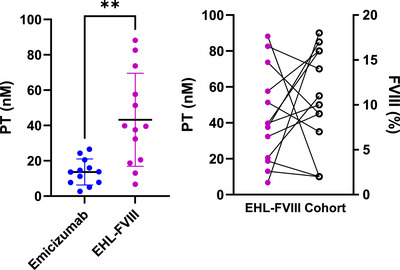
Peak thrombin is higher with EHL‐FVIII compared to EMI A). Dot plot comparing peak thrombin (PT, nM) between EMI and extended half‐life factor VIII (EHL‐FVIII) in individuals with haemophilia **A**. PT values were significantly higher in the EHL‐FVIII group (**p < 0.01), signifying enhanced thrombin generation. B). Scatter plot depicting PT (nM) across varying FVIII activity levels (%) within patients on EHL‐FVIII prophylaxis.

**FIGURE 3:**
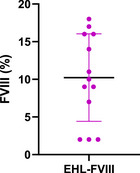
Factor VIII levels (%) within patients on EHL‐FVIII prophylaxis.

The ETP was significantly higher in the EHL‐FVIII cohort compared to the EMI cohort ([mean 418.9, SD 182.4 nanomolar‐minutes (nM.min)] vs. [164.9, SD 78.12 nM.min], *p* = 0.0002, Figure 1). After adjusting for age (<12 and ≥12) and BMI (overweight and obese vs normal weight), the observed trends in ETP were unchanged. PT was also greater in the EHL‐FVIII group ([mean 43.21, SD 26.34 nM] vs [mean 13.65, SD 7.40 nM *p* = 0.0010, respectively, Figure 2). Lag time and time to peak showed non‐significant trends favoring EHL‐FVIII. In the EHL‐FVIII prophylaxis group, the mean FVIII activity level was 10.23%, with a range of 2%–18% (Figure 3).

## 4 Discussion

Given that the reported FVIII‐equivalent activity is expected to be about 10%–30 % on EMI [7, 8] and our samples had a mean FVIII activity of 10.23% (2%–18%) on EHL‐FVIII therapies, these findings are striking and clinically relevant. Although ETP and PT levels are lower with EMI compared to EHL‐FVIII therapies, multiple clinical trials have demonstrated EMI's effectiveness in preventing bleeds in people with haemophilia A (PwHA). This includes evidence from a meta‐analysis and subgroup analysis of intra‐patient comparisons in the HAVEN 3 trial, which showed favorable outcomes even against FVIII prophylaxis [9]. However, the percentage of patients achieving zero bleeds has varied between 55% and 77% [10, 11, 12]. While showing suitable efficacy, we and others have reported severe spontaneous muscle bleeds requiring admission in pediatric and adult patients on EMI prophylaxis in real‐world settings [13, 14]. In another study standard EMI regimens in some adults did not control all bleeds [15]. Schmitt et al. reported maintenance dose up‐titration in only 3.6% (24/675) due to suboptimal bleeding control [16]. Establishing a direct relationship with bleeding requires dedicated studies including clinical bleeding phenotypes. Our data should be interpreted as descriptive within the context of assay conditions and further studies are needed to investigate whether ETP correlates with clinical outcomes such as breakthrough bleeding, especially joint bleeds, and by extension, joint health.

Our thrombin generation data are generally comparable to, or in some cases lower than, those reported in the literature, with observed differences largely influenced by assay conditions such as reagent type and activation methods. Kizilocak et al. reported TG parameters in a small cohort (*N* = 10) of individuals with haemophilia A on EMI prophylaxis using PPP low reagent without additional triggers. Their reported ETP and PT values (250.79 nM*min and 11.85 nM, respectively) closely mirror our findings, as we used a similar PPP low reagent approach [17]. However, it's important to note that PPP‐based assays may underestimate the hemostatic potential of EMI, which depends on the presence of platelets for full activity [18].

In contrast, the HAVEN 1 trial reported higher TG values in older patients (median age 29 years, range 12–75) on EMI prophylaxis, with a mean PT height of 108.7 nM (95% CI, 46.3–171.1) at week 72 of initiating EMI [7]. Their assay included FXIa as an additional trigger, which likely contributed to the elevated TG measurements. This reinforces the impact of assay conditions on the resulting hemostatic readouts.

These differences underscore the unique pharmacodynamics of EMI, a bispecific monoclonal antibody that bridges activated FIX and FX to mimic the cofactor function of FVIIIa. Unlike FVIIIa, EMI does not bind to phospholipid surfaces, such as activated platelets, and instead depends on platelets to bring FIXa and FX into proximity during thrombin generation. Studies employing platelet‐rich plasma (PRP) or FXIa spiking have demonstrated significantly greater TG responses, highlighting the importance of platelet presence in accurately assessing EMI's hemostatic potential [18].

Taken together with published literature, the lower TG parameters observed may be consistent with methodological influences rather than reduced EMI activity. PPP‐based assays without platelet involvement or additional triggers, such as those used in other studies, have shown to attenuate EMI‐mediated thrombin generation because its cofactor function relies on platelet‐driven colocalization of FIXa and FX‐a mechanism fundamentally distinct from FVIII. Thus, while our findings remain interpretable and comparable within the assay framework used, comparisons between EMI and EHL‐FVIII should be made with appropriate caution, and further work is needed to establish assay conditions that more fully reflect EMI's unique pharmacodynamics.

Limitations of our study include its single‐center and cross‐sectional design, which preclude the assessment of longitudinal changes in thrombin generation potential. The addition of clinical and patient reported outcomes would have strengthened our conclusions but at this pilot stage, our primary purpose was to compare surrogate markers of efficacy. Moreover, the FVIII activity assay used in our study differed from the one employed in clinical trials evaluating FVIII levels with ALTUVIIIO. Nonetheless, a laboratory field study of ALTUVIIIO demonstrated that our assay (PTT‐A reagent) yielded comparable results to the actin FSL reagent used in the pivotal trials [19]. Additionally, while FVIII levels were directly measured in this study, EMI concentrations were not assessed, however all participants were receiving stable prophylaxis and had no clinical signs suggestive of inadequate drug exposure. Although EMI plasma levels have been evaluated in previous studies to better understand pharmacokinetics and correlate with bleed protection (e.g., the HAVEN trials), such measurements were not incorporated into our analysis, representing a potential limitation in fully characterizing treatment exposure. Lastly, a key consideration is that our study did not include thrombin generation reference ranges from healthy controls or untreated pediatric patients with moderate/SHA, as their recruitment and sampling were not part of the study's objectives and were therefore not performed. Interpretation of absolute thrombin generation parameters is limited, particularly in the absence of established age‐specific pediatric reference ranges. The observed TG values should therefore be interpreted cautiously. Future studies incorporating healthy pediatric controls and untreated haemophilia A cohorts will be important to establish reference ranges and better contextualize TG findings in this population. Furthermore, TGA parameters are known to exhibit considerable inter‐individual and inter‐laboratory variability, even when the same reagents are used. This inherent variability may partially account for the lower peak and ETP values observed relative to previously published reports. These factors should be considered when interpreting the data, and future studies incorporating these comparator groups will help enhance contextual interpretation.

Moreover, thrombin generation parameters are known to vary with age, particularly in infants [20]. In our cohort, although EMI group included younger children, most participants were older than 2 years, when thrombin generation is relatively stable. Nevertheless, we acknowledge that age‐related differences may contribute modestly to inter‐individual variability, and this should be considered when interpreting our results.

Lastly, we note that individual TG results sometimes deviate from expected correlations with FVIII:C. For example, one patient with FVIII:C of 2% demonstrated a PT > 80 nM and ETP > 600 nM.min, whereas another patient with FVIII:C of 18%, had the lowest TG parameters. These discrepancies likely reflect a combination of biological variability, including differences in plasma composition, and technical limitations of the assay, such as a sensitivity to pre‐analytical handling and trigger conditions. Such variability underscores the need for cautious interpretation of individual thrombin generation results.

The strengths of our study include that we are providing real‐world patient data that evaluates and compares thrombin generation in pediatric patients receiving different therapeutic approaches, providing insights beyond controlled trial settings. By examining various novel therapies, our study provides key thrombin generation data across different treatment strategies for haemophilia A treatment. Moreover, most thrombin generation studies focus on adults, and our pediatric cohort provides unique insights into understanding age‐specific responses, which may influence treatment recommendations.

## 5 Conclusion

Pediatric patients on EHL‐FVIII prophylaxis, with mean trough FVIII levels around 10%, demonstrated higher ETP and PT in vivo when measured in PPP, compared to those receiving EMI prophylaxis. These findings reveal important biochemical differences between prophylactic regimens and support the need for further systematic studies using TGA with different triggers and in platelet‐rich plasma. Additional research is needed to clarify the clinical relevance of these differences, particularly regarding breakthrough bleeding and long‐term joint outcomes.

## Author Contributions

J.G. conceived and designed the study, analyzed the data, interpreted the results, and drafted the manuscript. S.G.Y. contributed to paragraph in methods section, interpreted the results, and critically edited the manuscript. R.S. interpreted results and critically edited manuscript. G.Y. interpreted the results and critically edited the manuscript. A.Z. designed the study, analyzed the data, interpreted the results, and critically edited the manuscript.

## Funding

The authors have nothing to report.

## Ethics Statement

The study was conducted in accordance with the Declaration of Helsinki and the applicable International Council for Harmonisation of Technical Requirements for Pharmaceuticals for Human Use Guideline for Good Clinical Practice.

## Conflicts of Interest

Jessica Garcia MD, has received honoraria for participating in scientific advisory board panels for Bayer, Genentech/Roche, Octapharma and Sanofi Genzyme, honoraria for speaking from Octapharma and Sanofi and received a 2025 HTRS/Novo Nordisk Clinical Scholar Award in Hemophilia and Rare Bleeding Disorders from the Hemostasis and Thrombosis Research Society (HTRS), which was supported by an educational grant from Novo Nordisk Inc. Sean Yates MD, and Ravi Sarode MD, have no conflicts to disclose. Guy Young MD, has disclosed that he has received consulting fees from Alnylam, ASC Biotherapeutics, Bayer, BioMarin, CSL Behring, Genentech/Roche, Hema Biologics, Hemab, Novo Nordisk, Octapharma, Pfizer, Sanofi Genzyme, Spark, and Takeda, and funds for research support from Sanofi. Ayesha Zia MD, has received honoraria for participating in scientific advisory board panels for Sanofi, Star Therapeutics and CSL Behring.

## Data Availability

The data that support the findings of this study are available on request from the corresponding author. The data are not publicly available due to privacy or ethical restrictions.

## References

[1] J. Oldenburg , “Optimal Treatment Strategies for Hemophilia: Achievements and Limitations of Current Prophylactic Regimens,” Blood 125, no. 13 (2015): 2038–2044, 10.1182/blood-2015-01-528414. 25712992

[2] A. Srivastava , E. Santagostino , A. Dougall , et al., “WFH Guidelines for the Management of Hemophilia, 3rd Edition,” Haemophilia 26 Suppl 6 (2020): 1–158, 10.1111/hae.14046. 32744769

[3] M. C. Pelland‐Marcotte and M. D. Carcao , “Hemophilia in a Changing Treatment Landscape,” Hematology Oncology Clinics of North America 33, no. 3 (2019): 409–423, 10.1016/j.hoc.2019.01.007. 31030810

[4] E. Santagostino , M. E. Mancuso , A. Tripodi , et al., “Severe Hemophilia With Mild Bleeding Phenotype: Molecular Characterization and Global Coagulation Profile,” Journal of Thrombosis and Haemostasis 8, no. 4 (2010): 737–743, 10.1111/j.1538-7836.2010.03767.x. 20102490

[5] A. Tripodi , V. Chantarangkul , M. Clerici , et al., “Thrombin Generation Assay for Testing Hemostatic Effect of Factor VIII Concentrates in Patients With Hemophilia A and Inhibitors: In Vitro Results From the PredicTGA Study,” Thrombosis Research 174 (2019): 84–87, 10.1016/j.thromres.2018.12.007. 30579150

[6] H. C. Hemker , P. Giesen , R. Al Dieri , et al., “Calibrated Automated Thrombin Generation Measurement in Clotting Plasma,” Pathophysiology of Haemostasis and Thrombosis 33, no. 1 (2003): 4–15, 10.1159/000071636. 12853707

[7] C. Schmitt , Adamkewicz JI , J. Xu , et al., “Pharmacokinetics and Pharmacodynamics of Emicizumab in Persons With Hemophilia A With Factor VIII Inhibitors: HAVEN 1 Study,” Thromb Haemost 121, no. 3 (2021): 351–360. 33086400 10.1055/s-0040-1717114 PMC7895541

[8] H. Kizilocak , E. Marquez‐Casas , J. Malvar , R. Carmona , and G. Young , “Determining the Approximate Factor VIII Level of Patients With Severe Haemophilia A on Emicizumab Using in Vivo Global Haemostasis Assays,” Haemophilia 27, no. 5 (2021): 730–735, 10.1111/hae.14359. 34115433

[9] A. Reyes , C. Révil , M. Niggli , et al., “Efficacy of Emicizumab Prophylaxis Versus Factor VIII Prophylaxis for Treatment of Hemophilia A Without Inhibitors: Network Meta‐Analysis and Sub‐Group Analyses of the Intra‐Patient Comparison of the HAVEN 3 Trial,” Current Medical Research and Opinion 35, no. 12 (2019): 2079–2087, 10.1080/03007995.2019.1649378. 31355677

[10] J. Oldenburg , J. N. Mahlangu , B. Kim , et al., “Emicizumab Prophylaxis in Hemophilia A With Inhibitors,” New England Journal of Medicine 377, no. 9 (2017): 809–818, 10.1056/NEJMoa1703068. 28691557

[11] G. Young , R. Liesner , T. Chang , et al., “A Multicenter, Open‐Label Phase 3 Study of Emicizumab Prophylaxis in Children With Hemophilia A With Inhibitors,” Blood 134, no. 24 (2019): 2127–2138, 10.1182/blood.2019001869. 31697801 PMC6908828

[12] J. Mahlangu , J. Oldenburg , I. Paz‐Priel , et al., “Emicizumab Prophylaxis in Patients Who Have Hemophilia A Without Inhibitors,” New England Journal of Medicine 379, no. 9 (2018): 811–822, 10.1056/NEJMoa1803550. 30157389

[13] J. Garcia , M. Hammer , and A. Zia , “Serious Bleeds in Pediatric Hemophilia A Patients on Emicizumab Prophylaxis,” 2023: Research and Practice in Thrombosis and Haemostasis (In press) 7, no. 8: 102238. 10.1016/j.rpth.2023.102238 PMC10694589 38053983

[14] G. Batsuli , A. P. Wheeler , A. C. Weyand , R. F. jr Sidonio , and G. Young , “Severe Muscle Bleeds in Children and Young Adults With Hemophilia A on Emicizumab Prophylaxis: Real‐World Retrospective Multi‐Institutional Cohort,” American Journal of Hematology 98, no. 10 (2023): E285–E287, 10.1002/ajh.27039. 37471655

[15] S. Levy‐Mendelovich , T. Brutman‐Barazani , I. Budnik , et al., “Real‐World Data on Bleeding Patterns of Hemophilia A Patients Treated With Emicizumab,” Journal of Clinical Medicine 10, no. 19 (2021): 4303, 10.3390/jcm10194303. 34640320 PMC8509656

[16] C. Schmitt , M. E. Mancuso , T. Chang , et al., “Emicizumab Dose Up‐Titration in Case of Suboptimal Bleeding Control in People With Haemophilia A,” Haemophilia 29, no. 1 (2023): 90–99, 10.1111/hae.14679. 36271487 PMC10091821

[17] H. Kizilocak , E. Marquez‐Casas , C. Phei Wee , J. Malvar , R. Carmona , and G. Young , “Comparison of Bypassing Agents in Patients on Emicizumab Using Global Hemostasis Assays,” Haemophilia 27, no. 1 (2021): 164–172, 10.1111/hae.14213. 33245833

[18] J. Muller , M. Büchsel , O. Oleshko , et al., “Laboratory Monitoring in Patients Receiving Emicizumab,” Hamostaseologie (2025):Epub ahead of print. 10.1055/a-2687-0107 41067253

[19] S. W. Pipe , Efanesoctocog Alfa Activity Assessment With One‐Stage Clotting (OSA) and Chromogenic Substrate (CSA) Factor VIII Assays., in EAHAD 2023 (Manchester, England) (2023).

[20] S. Gassiot , A. Ruiz‐Llobet , W. Suleman , E. Sarrate , and R. Berrueco , “Thrombin Generation in Children Using ThromboScreen Reagent Kit With ST Genesia‐A Pilot Study,” Int J Lab Hematol 43, no. 6 (2021): 1612–1619, 10.1111/ijlh.13668. 34323010

